# Absence/presence calling in microarray-based CGH experiments with non-model organisms

**DOI:** 10.1093/nar/gku343

**Published:** 2014-04-25

**Authors:** Martijs J. Jonker, Wim C. de Leeuw, Marino Marinković, Floyd R. A. Wittink, Han Rauwerda, Oskar Bruning, Wim A. Ensink, Ad C. Fluit, C. H. Boel, Mark de Jong, Timo M. Breit

**Affiliations:** 1 MicroArray Department & Integrative Bioinformatics Unit (MAD-IBU), Swammerdam Institute for Life Sciences (SILS), Faculty of Science (FNWI), University of Amsterdam (UvA), 1098 XH, Amsterdam, the Netherlands; 2 Netherlands Bioinformatics Centre (NBIC), 6525 GA, Nijmegen, the Netherlands; 3 Department of Aquatic Ecology and Ecotoxicology, Institute for Biodiversity and Ecosystem Dynamics (IBED), University of Amsterdam, Amsterdam, the Netherlands; 4 Medical Microbiology, University Medical Center Utrecht, Utrecht, the Netherlands

## Abstract

Structural variations in genomes are commonly studied by (micro)array-based comparative genomic hybridization. The data analysis methods to infer copy number variation in model organisms (human, mouse) are established. In principle, the procedures are based on signal ratios between test and reference samples and the order of the probe targets in the genome. These procedures are less applicable to experiments with non-model organisms, which frequently comprise non-sequenced genomes with an unknown order of probe targets. We therefore present an additional analysis approach, which does not depend on the structural information of a reference genome, and quantifies the presence or absence of a probe target in an unknown genome. The principle is that intensity values of target probes are compared with the intensities of negative-control probes and positive-control probes from a control hybridization, to determine if a probe target is absent or present. In a test, analyzing the genome content of a known bacterial strain: *Staphylococcus aureus* MRSA252, this approach proved to be successful, demonstrated by receiver operating characteristic area under the curve values larger than 0.9995. We show its usability in various applications, such as comparing genome content and validating next-generation sequencing reads from eukaryotic non-model organisms.

## INTRODUCTION

Microarray-based comparative genomic hybridization (aCGH) is widely used in biomedical applications and life sciences research to detect and analyze structural variation in genomes (1–5), and novel applications are constantly developed (6–8). Genomic structural variations include aberrations such as insertions and deletions (indels), duplications and other copy number variants (2). In conventional aCGH experiments, the probes on a microarray are designed to recognize specific target sequences from a reference genome (9). In an aCGH test, DNA from an unknown test genome and reference genome are labeled individually with a different fluorescent dye (i.e. dual channel/color) and hybridized together onto the microarray. Also single-color approaches are used in which the DNA from test and reference genome are labeled with the same fluorescent dye, but hybridized to individual microarrays. The fluorescence intensity signals from labeled DNA that hybridized to target probes in a spot on the microarray are processed and normalized. The difference between the intensity signals of each probe from the test and reference genome, expressed as log2 ratios, is analyzed to detect genomic alterations and aberrations (10). An increased log2 ratio represents a higher number of target sequences in the test genome compared to the reference genome. Conversely, a decrease indicates a lower number of identical target sequences in the test genome compared to the reference genome, an equal number of similar but non-identical target sequences in the test genome or both. Due to the complexity of eukaryotic genomes, the total signal of a microarray hybridization get diluted and makes aCGH data often quite noisy (11). Hence over the past years many statistical procedures have been developed to analyze these data (12).

The simplest procedure is to use a fold change cut-off. Thomas *et al.* (13) for instance classified log2 ratios greater than 1.15 as gain of target sequence and less than 0.85 as loss. Others assumed that normal log2 ratios are (approximately) normally distributed, and this distribution is then used for statistical inference. Hodgson *et al.* (14), for instance, fitted a mixture of three Gaussian distributions to a histogram of log2 ratios, representing a ‘normal’ component centered at 0, a ‘loss’ component centered at a mean less than 0 and a ‘gain’ component centered at a mean greater than 0. These methods infer single probes which has been shown to be trustworthy under stringent hybridization conditions (15). Nevertheless, over the years it has been established that inferring copy number from single probes is error prone. Thus, algorithms have been developed that use information from probes to target sequences that are adjacently located in the genome to identify larger structural-variant regions with more confidence. These algorithms take the probe-target order (or location) on the genome as input and are frequently based on smoothing methods or hidden Markov models (reviewed and compared by Lai *et al.* (16) and Dellinger *et al.* (12)). They are particularly useful for microarrays with probes designed with high genomic resolution based on well-annotated genomes. As such, these data analysis procedures are most applicable to so-called ‘model organisms’, such as mouse and human. On the other hand, aCGH experiments with ‘non-model organisms’ are less well formalized and the data analysis procedures are less well established. When the genome of the organism of interest is not (fully) sequenced, probes may be designed using transcriptome sequences (e.g. expressed sequence tags (ESTs) or sequencing reads), or using genome information from a closely related sequenced species or strain (17,18). The actual order of the probe targets in the genome under study may then be uncertain or even unknown. In such cases, the assumption that the genome of a non-model organism is similar to a reference genome of a related species may be wrong.

Hence, we present here an additional approach to copy number estimation that quantifies the presence or absence of a probe target in aCGH analysis (quantified absence/presence calling (QAPC)). The underlying principle is straightforward: probe intensity values are compared with the intensities of negative-control probes, which are known to have no targets in the control sample, to test the null hypothesis that a target sequence is absent in the test genome, or inversely with positive controls for present calling. It has in principle a resolution of a single probe (in this study ∼60 nucleotides). The aGCH QAPC method does not depend on the structural information of a reference genome: the genomic ordering of probe targets is irrelevant. The method was tested by analyzing the known genomic content of prokaryote *Staphylococcus aureus* (*S. aureus*) strain MRSA252. As for this strain it is known which probes do match the genome, this allows quantification of the error rates of the target sequence absence/presence calls. Together, three examples are presented to illustrate that this method can be used for various purposes: (i) to define a core genome without using the structural information of a reference genome, (ii) to compare the genomic content of multiple genomes, (iii) to validate next-generation sequencing (NGS) reads from eukaryotic non-model organisms.

## MATERIALS AND METHODS

### Samples and sample processing

The QAPC method was validated with aCGH analyses on bacterium *S. aureus*, zebrafish *Danio rerio (D. rerio)* and midges *Chironomus riparius* (*C. riparius*) and *Anopheles gambiae* (*A. gambiae*). Eleven *S. aureus* strains were used: MRSA252 (NCBI Reference Sequence: NC_002952.2), MSSA476 (NC_002953.3), Mu50 (NC_002758.2), MW2 (NC_003923.1), N315 (NC_002745.2), NCTC8325 (NC_007795.1), Newman (NC_009641.1), RF122 (NC_007622.1), S0385, USA300, WKZ1. The strains were grown overnight in Iscove's Modified Dulbecco's Medium (IMDM) (Invitrogen, Carlsbad, CA, USA) (19). The overnight cultures were diluted (1:7) in fresh pre-warmed IMDM and grown twice to mid-log phase culture (a A660nm of 0.5). The second mid-log phase culture was diluted to an A660nm of 0.3 with pre-warmed IMDM and directly transferred to fresh pre-warmed IMDM to obtain an A660nm of 0.03. Samples for DNA extraction were taken at 4 h post-inoculation. The zebrafish adults were kept under standard conditions. Two adults, one male and one female, were sacrificed for DNA analyses in six replicates each. The growth conditions of the midges are described in detail by Marinkovic *et al.* (20). DNA was extracted from 30 pooled fertilized *C. riparius* egg ropes and 30 pooled unfed *A. gambiae* adults. The DNA from the bacteria was purified using the NucleoSpin®’ Tissue kit (Macherey-Nagel; 740 952 50) according to manufacturer's protocol. The DNA from the zebrafish adults and the midges was extracted using a CTAB DNA extraction method (20). DNA yield was measured using a NanoDrop ND-1000 (Nanodrop Technologies, Wilmington, DE, USA).

### Array design

Three different microarrays were used in this study: (i) a *S. aureus* multi-strain tiling microarray, (ii) a *D. rerio* 3’-biased expression microarray and (iii) a *C. riparius* transcriptome validation microarray. The *S. aureus* microarray (12 × 135K NimbleGen) was designed using publicly available genomic sequences of 18 strains, 19 plasmids and 6 phages associated with *S. aureus*. Probes were designed to genome target sequences each 40 base pairs on alternating strands for the first sequence. For each next sequence only oligos were designed for regions, which were not probed by previously designed oligos (bit-score > 80). Using these parameters, 121 901 probes were generated. Additionally, the *S. aureus* microarray also contains 5000 negative-control probes of random sequences that do not target any genome or transcriptome in Entrez (21).

Details for the *D. rerio* microarray (12 × 135K Nimblegen) can be found in Rauwerda *et al.* (22). It is a 3’ biased expression microarray with 63 751 target probes and 7.742 probes with random sequences not targeting the *D. rerio* genome, which were used as negative-control probes.

The *C. riparius* transcriptome validation microarray (1 × 1M Agilent Technologies) was designed against 1 540 849 transcriptome 454 NGS reads (20). The complete design consisted of two collections of probes. First, 60-mers probes were designed to target all the *C. riparius* NGS reads at least once, but possibly three times or more. This resulted, after testing for cross-hybridization and validating for adequate GC-content (20–60%), in 843 837 *C. riparius* NGS-read probes. Second, probes were designed against publicaly available ESTs of *C. riparius*, the other *Chironomus* species and the closely related dipteran species *A. gambiae*, *Anopheles darlingi, Anopheles funestus, Aedes aegypti and Culex quinquefasciatus*, that were not already targeted by the *C. riparius* NGS-read probes from the first step. This step yielded an additional 75 984 probes. The design was finalized by including 40 000 negative-control probes that covered a wide GC-content range. In addition, the total collection of NGS reads was blasted against the human genomic and transcript data available at NCBI. As match score we used (match length * identity)/read length, and a score higher than 95 ( = bit-score 119) was considered a perfect match.

### Amplification, labeling, hybridization and scanning

Genomic DNA was amplified and labeled by strand displacement amplification. Two hundred nanogram genomic DNA was combined with 5 μg random octamers (Biolegio). The DNA was partly fragmented by heat at 99°C for 10 min and allowed to cool on an ice/water bath for 5 min. The DNA/octamer mix was made on ice to 50 mM Tris-Cl (pH 7.5), 5 mM MgCl2, 1 mM DTT, 1 mM dGAC (GE Healthcare), 0.6 mM dTTP (GE Healthcare), 0.4 mM aminoallyl dUTP (TriLink Biotechnologies) and finally 10U Klenow exo- (Jena Bioscience) was added. After gentle vortexing, the samples were place at 37°C for 8 h and subsequently incubated for 15 min at 75°C to inactivate the enzyme. Each reaction was made to 1.5 M sodium acetate (pH 5.2) and the amplified DNA was purified with the QIAquick PCR Purification Kit (Qiagen) according to the manufacturer's instructions with the exception that seven volumes of binding buffer were used instead of five to increase the recovery of smaller fragments. Concentrations of amplified products were measured on the NanoDrop ND-1000 (Thermo Scientific) and quality assessed on the BioAnalyzer (Agilent Technologies) with the DNA 1000 Kit (Agilent Technologies). From each sample 5 μg was taken, dried down and dissolved in 50 mM carbonate buffer (pH 8.5). Individual vials of Cy3/Cy5 from the mono-reactive dye packs (GE Healthcare) were dissolved in 200 μl DMSO. To each sample, 10 μl of the appropriate CyDye dissolved in DMSO was added and the mixture was incubated for 1 h. Reactions were quenched with the addition of 5 μl 4M hydroxylamine (Sigma-Aldrich). This mixture was made to 0.75M Ammonium acetate and 0.2 μg/μl glycogen (Ambion). After mixing 2.5 vol. of 100% EtOH was added and mixed. All samples were incubated for at least 60 min at −20°C to enhance precipitation and centrifuged for 30 min at 13.000xg. The supernatants were carefully removed by pipetting and 1 vol. of 80% EtOH was added to the pellets. After brief vortexing, the labeled DNA was pelleted once more by centrifugation for 10 min at 13.000xg. The supernatants were removed by pipetting and the pellets were dried by vacuum concentrating. Final labeled pellets were dissolved in 30 μl H2O and the yield and CyDye incorporation were measured with the NanoDrop ND-1000.

Each hybridization mixture was made up from 1 μg test material (Cy3) and 1 μg reference material (Cy5). The *S. aureus* experiments were performed according a common reference design, with all 11 strains as test samples in triplicates, and MRSA252 as common reference. The *D. rerio* experiments also with 12 individuals as test samples and a pool as common reference. *C. riparius* (Cy3) was directly hybridized against *A. gambiae* (Cy5). The combined samples were dried and 1.98 μl of water was added. The hybridization cocktail was made according to the manufacturer's instructions (NimbleGen microarrays User's Guide—Gene Expression microarrays Version 5.0, Roche NimbleGen). 5.22 μl from this mix was added to each sample. The samples were incubated for 5 min at 95°C and 5 min at 42°C prior to loading. Hybridization samples were loaded onto the microarray and hybridized for 20 h at 42°C with the NimbleGen Hybridization System 4 (Roche NimbleGen). Afterwards, the slides were washed according to the NimbleGen microarrays User's Guide—Gene Expression microarrays Version 5.0 and scanned in an ozone-free room with an Agilent DNA microarray scanner G2565CA (Agilent Technologies). Feature extraction was performed with NimbleScan v2.6 (Roche NimbleGen). All microarray probe designs and data have been submitted to the Gene Expression Omnibus, and have accession numbers GSE53449 and GSE52122.

### Data analysis

All slides were subjected to a set of quality control checks, i.e. visual inspection of the scans and pseudo-color plots, consistence of performance across replicate samples and visual inspection of pre- and post-normalized data with box-and-whisker, ratio-intensity and principal component plots. Only samples that met the quality criteria were subjected to data analysis using the R environment (23).

Before applying a formal procedure for absence calls, the raw data were examined to determine whether a procedure based on negative-control probes had any potential to yield reliable results. Using thousands of probes designed to be negative controls, and probes designed to perfectly match target sequences in the genome, we investigated if probe intensity could be used to determine whether target sequences were present in genomic samples from *S. aureus* strain MRSA252 and *D. rerio*. This was analyzed as a standard binary classification problem based on receiver operating characteristic (ROC) curves (ROCR; (24)).

The QAPC method proceeds by comparing the microarray hybridization signals of the test genome, with the signals of the positive controls, which can be performed using a known genome. Basically, intensities of probes that are known to match the control genome and probes that are known not to match this genome are used to infer the probe intensities of the unknown test sample. To make the hybridization intensities of the control and the test genomes equivalent, an adjusted quantile normalization procedure was applied (25). It performs quantile normalization across a set of invariant features and then for each sample fills in the remaining features by linear interpolation. In short, the expression matrix (*X*) is reduced to the invariant set:

\begin{equation*} X = [x_{n1} ,...,x_{n\left| N \right|} ,x_{m1} ,...,x_{m\left| M \right|} |n_i \in N,m_j \in M] \end{equation*}

which is subjected to quantile normalization. Let *j’* and *j*’ be two features in *N*∪*M*, that are the nearest invariant features to *x_ij_*, such that *x_ij_’* < *x_ij_* < *x_ij_*’. Let *y*_1_ and *y*_2_ be the quantile normalized values. Let there be *a* values between *x_ij_’* and *x_ij_*, and *b* values between *x_ij_’* and *x_ij_*’ on microarray *i*. Then

\begin{equation*} x_{ij}^{\rm norm} = y_1 + \frac{{a + 1}}{{a + b + 1}}\left( {y_2 - y_1 } \right) \end{equation*}

is the normalized value of features *j* on microarray *i*. The invariant set was defined as the set containing those features with a less than or equal to one log2-fold change between the test and the known genome.

When the intensity data from the control and the test genomes are made equivalent, they can be compared. The data from the control genome is used to calculate a *P*-value for absence, given intensity *x*, based on the perfect-match probes (as positive controls: present) and the predefined set of negative controls (absent), using standard Bayes rule:

\begin{eqnarray*} &&P\left({\rm absent|\it x} \right) = \\ &&\frac{{P\left({x|\rm absent} \right)P\left({\rm absent} \right)}}{{P\left({x|\rm absent} \right)P\left({\rm absent} \right) + P\left({x|\rm present} \right)P\left({\rm present} \right)}} \end{eqnarray*}

The value for *P*(absent|*x*) is directly applied to the data from the test genome. Obviously, *P*(present|*x*) = 1−*P*(absent|*x*), and the researcher should decide on a *P*-value cut-off based the certainty with which conclusions need to be drawn. For instance, a high certainty that a probe target is present is achieved when the cut-off is set to *P*(present|*x*) > 0.99. The equation above can be modeled in many ways, depending on the assumptions concerning the intensity distributions of the positive- and negative-control probes. We used empirical distributions to represent *P*(*x*|absent) and *P*(*x*|present), and we used two parametric approaches and a non-parametric approach. The parametric approaches are (i) fitting a shifted log-normal distribution to model *P*(*x*|absent) and a left-truncated Gaussian distribution to model *P*(*x*|present) or (ii) fitting Gaussian distributions to model both *P*(*x*|absent) and *P*(*x*|present). A third method without distributional assumptions, useful in case of a lack of fit, is to quantify *P*(*x*|absent) and *P*(*x*|present) using density estimation whilst optimizing the bandwidth for conditional density estimates (26). This procedure was found to be too computationally expensive to be practical for large data sets, so we applied it after using a data reduction scheme (Supplementary Methods), yielding approximate significance values. *q*–*q* plots were used to decide which approach to use. A good choice of the empirical distributions is crucial, and a decision protocol is supplied in the Supplementary information. The QAPC measure can also be calculated without a control genome. With strong distributional assumptions the mixture of signals from probes without and with target can be de-convoluted using an expectation maximization algorithm (Supplementary Methods).

Prior to data analysis, microarray data are usually pre-processed and subjected to within and between microarray normalization to correct for sources of systematic variation. We investigated whether within and between microarray normalization improves the QAPC method. Values for *P*(absent|*x*) were calculated from raw data, within microarray sequence-normalized data, and between microarray LOESS-normalized data. Sequence-based normalization is generally recommended for high density aCGH analysis and was performed according to Naef and Magnasco (27), using the model:

\begin{equation*} \hat E_i = \sum\limits_{k = 1}^m {\bar E_{i,k} + \bar E} \end{equation*}

where *Ê_i_* indicates predicted log2 intensity due to ubiquitous hybridization for feature *i*, *Ē_i,k_* indicates the mean log2 intensity of probes having the same nucleotide at position *k* and *Ē* is the overall average log2 intensity. This model has been suggested and investigated for tiling microarrays by Munch *et al.* (28) and Royce *et al.* (29). Local polynomial regression (LOESS) normalization was applied to signals of each individual microarray against the signals of a reference median microarray. As genomes can substantially differ, we implemented a robustified LOESS normalization procedure that used a bandwidth abs(*M*) = 1, which means that the LOESS line was based on the probes with intensity differences less than one log2-fold change between the sample and the reference median microarray. We first performed an initial fit using probes abs(*M*) < 1 between the sample and the reference median array. The residuals (*ϵ_i_*) were computed and used to calculate probe-specific weights: *w_i_* = 1, for |*ϵ_i_*| < 1, and *w_i_* = 0, for |*ϵ_i_*| > 1. These steps were iterated which was stopped after four iterations or if

\begin{equation*} \frac{{\sum {\left( {\hat M_i^{\rm iter} - \hat M_i^{\rm iter - 1} } \right)^2 } }}{n} {<} 10^{ - 4} \end{equation*}

The performance of the absence calls was tested by analyzing the genomic content of a sequenced *S. aureus* strain MRSA252. For this strain it is known for which probes a target sequence is present in the genome, enabling quantification of the error rates. Analyses on different strains of *S. aureus* and the midges *C. riparius* and *A. gambiae* were performed to demonstrate applications.

## RESULTS

### Using probe intensity for absence/presence calling of a target

**Figure 1.**
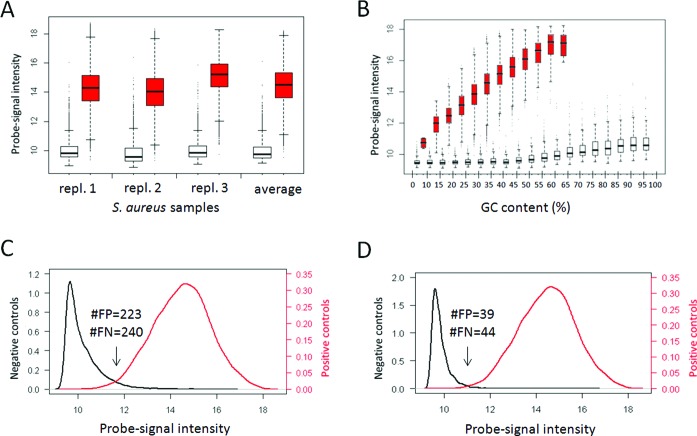
Evaluating *S. aureus* negative and positive controls. (**A**) box-and-whisker plots showing the signal intensity distributions of negative-control (white) and perfect-match probes (red) of three replicate *S. aureus* MRSA252 samples and based on their averaged signal intensity. (**B**) box-and-whisker plots showing the intensity distributions of negative-control probes (white) and perfect-match probes (red) of the averaged intensity in GC bins. (**C**) Density plot indicating the signal intensity distribution of the negative control (black) and the perfect-match probes (red). The numbers indicate the minimum number of false positives (FP) and false negatives (FN) absence calls, obtained at a log2 intensity of 11.3. (**D**) As C, but after selecting the probes with GC content between 10% and 65%. The numbers are obtained at a log2 intensity of 10.8.

To determine to what extent probe intensity indicates the presence of a probe target, we first investigated raw aCGH data from prokaryote *S. aureus* and eukaryote *D. rerio*. The *S. aureus* multi-strain tiling microarray was designed with 5000 negative-control probes and 46,588 perfect-match probes to *S. aureus* strain MRSA252. This microarray was used in an aCGH experiment using three replicate DNA samples of the *S. aureus* strain MRSA252 (Figure 1). As expected, the negative-control probes showed substantial lower signal intensities than the perfect-match probes (Figure 1A), although there is some overlap between the two distributions (Figure 1C). We determined a minimum error of 463: with 223 perfect-match probes called absent and 240 negative controls were called present at a log2 intensity of 11.3, equivalent to an accuracy of 0.9910 and an area under the curve (AUC) of 0.9978.

However, it turned out that there was a strong correlation between the GC content of the probes and their signal intensity (Figure 1B). In contrast to the perfect-match probes with a maximal GC content of 65%, the negative-control probes have GC content up to 100% and the signal intensities of the negative-control probes are increasing from a GC content ≥ 55%. At the same time, perfect-match probes with a GC content < 10% showed a quite low signal. So, the overlap between the negative controls and the perfect-match probes can largely be explained by the high GC content of many negative-control probes and the low GC content of a few perfect-match probes (Figure 1A). Based on these results, we decided to only use probes with GC content higher than 10% and lower than 65%; 2811 negative-control and 46 582 perfect-match probes remained. This resulted in a lower overlap between the intensity distributions of the negative-control and perfect-match probes show. The minimum error drops to 83 (39 false positive and 44 false negative absence calls) at a log2 intensity of 10.8, which is equivalent to an accuracy of 0.9983 and an AUC of 0.9994. The classification performance of the three individual replicate measurements and the average measurement shows that, when the probe intensity is used as a proxy for the presence of a probe target, the error rates are low and the precision and the accuracy are high (Table 1). The AUC is higher than 0.999 in all replicates, indicating that a large separation between the negative-control and perfect-match probes can be achieved with a high consistency.

**Table 1.** Confusion table values showing classification performance

	*S. aureus*				*D.rerio*		
	repl. 1	repl. 2	repl. 3	Mean signals	Sample 1	Sample 2	Mean signals^a^
True positives	2734	2649	2773	2766	5408	5807	5784
False positives	41	87	22	39	1503	1241	1293
True negatives	46 542	46 496	46 561	46 544	54 506	54 768	54 716
False negatives	76	161	37	44	1,363	964	987
True positive rate	0.9730	0.9427	0.9868	0.9843	0.7987	0.8576	0.8542
False positive rate	0.0009	0.0019	0.0005	0.0008	0.0268	0.0222	0.0231
True negative rate	0.9991	0.9981	0.9995	0.9992	0.9732	0.9778	0.9769
False negative rate	0.0270	0.0573	0.0132	0.0157	0.2013	0.1424	0.1458
Precision	0.9852	0.9682	0.9921	0.9861	0.7825	0.8239	0.8173
Accuracy	0.9976	0.9950	0.9988	0.9983	0.9543	0.9649	0.9637
Area under the curve	0.9992	0.9978	0.9997	0.9994	0.9760	0.9829	0.9823

^a^Mean of 2 biological x 6 technical replicates (Supplementary information).

To check this approach with an eukaryotic genome, a similar aCGH analysis was performed on the genome of *D. rerio* using a microarray with 7742 negative-control and 56 009 perfect-match probes. The perfect-match probes were designed with a GC content ranging from 33% until 57%. To align the negative-control probes, 6771 negative-control probes within the same CG range were selected for the subsequent analyses. An experiment using 2 × 6 replicate *D. rerio* samples showed that the negative-control probes have a consistently lower intensity than the perfect-match probes, but again there is overlap between the two intensity distributions (Supplementary Figure S1). The overlap is larger than observed for *S. aureus* (Table 1), but the AUC of ∼0.98 indicates a good separation between the signal intensities of the negative-control and perfect-match probes, a steady observation among the replicate measurements (Table 1, Supplementary Table S1).

**Figure 2.**
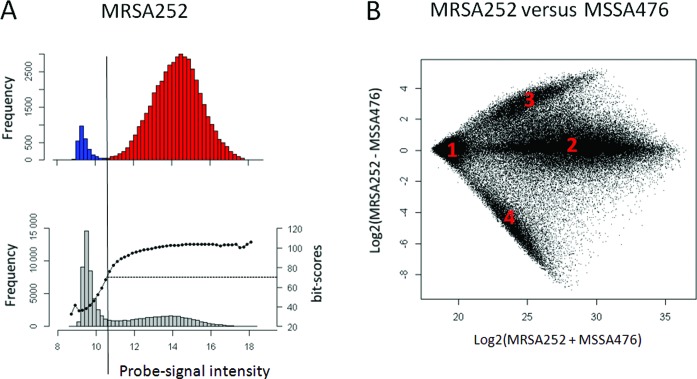
Present/absence calling in *S. aureus* by means of probe-signal intensities of negative and positive controls. (**A**) Histogram showing the intensity distribution of negative-control probes (top panel, blue), perfect-match probes (top panel, red) and remaining probes on the microarray (bottom panel, grey) with the average bit-scores of each bin (dotted black line, bottom panel) for *S. aureus* strain MRSA252. (**B**) A ratio-intensity plot showing the log2 ratio against the log2 sum of the MRSA252 and MSSA476 probe intensity signals. The four groups, indicated in the graph, are discussed in the main text.

To see if this approach could be helpful in determining the absence or presence of target sequences in test genomes, we analyzed all the remaining probes on the *S. aureus* multi-strain microarray that were designed for other strains than strain MRSA252. For this, we applied the minimal cut-off observed with the strain MRSA252, log2 intensity of 10.8 to all non-MRSA252 designed probes (Figure 2A). Many probes showed signal intensities equivalent to the negative controls, whereas other probes have higher signal intensities (Figure 2A, lower panel). Thus, there are probes without and with target sequence, respectively. As such, there should be a relation between the probe bit-score for the best match with the strain MRSA252 genome and the probe-signal intensity. Indeed, probes with a log2 signal intensity above 10.8 have an average bit-score of over 70 (Figure 2A, lower panel). This implies that the target sequences from the probes with a signal intensity below the cut-off are generally absent from the strain MRSA252 genome. To test this approach with another MRSA strain, we compared the MRSA252 probe intensities with the intensities of the probes resulting from a hybridization with the MSSA476 genome and identified four probe groups (Figure 2B): Group 1, probes which targets are absent in both the MRSA252 and MSSA476 genomes, as their intensities are low in both hybridizations; Group 2, probes which targets are present in both the MRSA252 and MSSA476 genomes, as their intensities are high in both hybridizations; Group 3, probes which targets are present in MRSA252, but absent in MSSA476, as their intensities are high after hybridizing the MRSA252 genome but low in MSSA476 and reversely Group 4, probes which targets are absent in MRSA252 but present in MSSA476. In the next section, we will show how these observations can be used to analyze unknown genomes.

### Statistical procedure for QAPC

The results from the raw data indicate that probe intensities can in principle be used to make present/absent calls, but a formal data analysis procedure is required to enable a genuine quantitative analysis. The QAPC procedure we propose here requires that microarrays are fitted with probes designed against a certain control genome and probes that can serve as negative controls. The data analysis procedure is based on comparing the data from the control genome with the data from the unknown test sample. The intensities of probes that are known to match the control genome and probes that are known not to match the control genome, i.e. negative controls, are used to infer the target status of the test sample. Absence/presence calls can then be quantified as described in Materials and Methods (plus Supplementary Methods). We propose the following procedure, which we call the QAPC method:

1. Normalize the data within and/or between microarrays, if removal of technical variability is required.
2. Select probes with a proper GC content (typically higher than 10% and lower than 65%).
3. Average the log2 probe intensities among the replicate microarrays.
4. Before comparing the control and the test genome, ensure that the intensities are equivalent, for instance by performing a robustified quantile normalization.
5. Use the control genome to calculate a *P*-value for absence/presence of a probe target sequence given a certain probe-signal intensity.
6. Use these *P*-values for a statistical inference of target sequences in the unknown genome.

**Figure 3.**
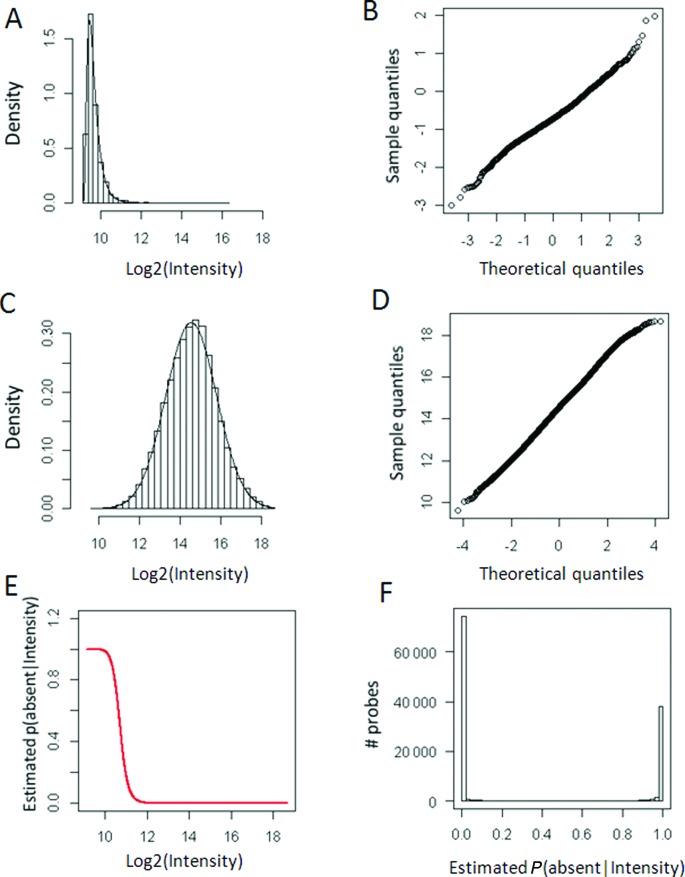
Goodness of fit and outcome of the empirical statistical modeling procedure. (**A**) Histogram and density plot showing the fit of the shifted log-normal distribution to the intensities of the negative-control probes. (**B**) Quantile–quantile plot showing the fit of the shifted log-normal distribution to the intensities of the negative-control probes. (**C**) Histogram and density plot showing the fit of the normal distribution to the intensities of the perfect-match probes. (**D**) Quantile-quantile plot showing the fit of the normal distribution to the intensities of the perfect-match probes. (**E**) Bayesian *P*-values plotted against probe-signal intensity. (**F**) Histogram showing the distribution of the *P*(absent|intensity) values.

To determine the performance of the above described procedure, we analyzed strain MRSA252 using the genome of sequenced *S. aureus* strain MSSA476 as control. To examine the robustness of the approach, we initially omitted step 1. The filtering for the probes with the appropriate GC content yielded 2811 negative controls and 41 119 positive controls (step 2). The genomes of MRSA252 and MSSA476 were analyzed in triplicates that were averaged (step 3). After the quantile normalization (step 4), the *P*-values were calculated by empirically fitting the shifted log-normal distribution onto the negative-control probes and the left-truncated normal distribution onto the perfect-match, positive control, probes (step 5, Figure 3A–D). Estimation of absence *P*-values at certain fluorescence intensity revealed that the probability of absence of a target is low when the probes have log2 signal intensities higher than 12 (Figure 3E). The distribution of *P*-values showed that probes either have a very high or a very low *P*-value (Figure 3F). When the *P*-value cut-off is set to *P* ≤ 0.01, we found that 73 501 probes have a target, given their intensity, and 48 176 targets are called absent. It is noteworthy that the quantile–quantile plots indicate that the probability density functions, though based on empirical considerations, fit the data (Figure 3B and D). To determine the actual performance of the statistical analysis, we turned to the strain MRSA252, as for this strain the probes that match the genome are known. For this analysis, we presumed that the 30 351 probes with bit-scores ≤ 40 should give absence calls and that the 46 582 perfect-match probes with bit-scores 119 should give no absence calls. Using raw data, low numbers of false positive (601) and false negative (69) calls were made, hence true positives and true negatives show low error rates (∼1%) and an encouraging AUC value of 0.9997 (Table 2).

**Table 2.** Confusion table values to show the classification performance of probes with bit-scores ≤ 40 and probes with bit-score 111 when compared to the genome of *S. aureus* strain MRSA252 after applying the statistical procedure (*P*(absent|intensity) < 0.01) to raw data, data after sequence normalization and data after LOESS normalization

	Raw data	Seq. norm	LOESS norm
True positives	30 282	30 284	30 284
False positives	601	82	629
True negatives	45 981	46 500	45 953
False negatives	69	67	67
True positive rate	0.9977	0.9978	0.9978
False positive rate	0.0129	0.0018	0.0135
True negative rate	0.9871	0.9982	0.9865
False negative rate	0.0023	0.0022	0.0022
Precision	0.9805	0.9973	0.9797
Accuracy	0.9913	0.9981	0.9910
Area under the curve	0.9997	0.9998	0.9997

**Figure 4.**
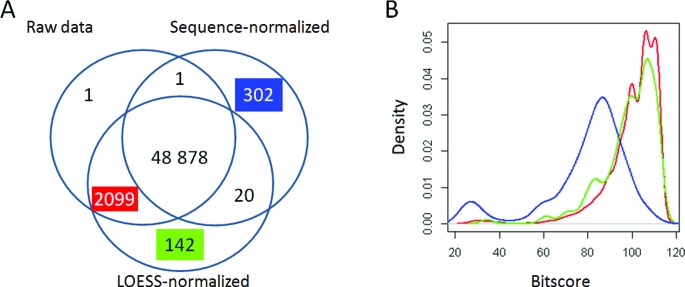
Comparing normalization approaches in QAPC. (**A**) Venn diagram of the absence-called probe sets, based on raw, sequence-normalized and LOESS-normalized data. (**B**) Density plot showing the distributions of bit-scores of three overlaps from the Venn diagram. As compared to the strain MRSA252 genome: the probes called absent after analyzing the sequence-normalized data (302, blue), the LOESS-normalized data (142, green) and raw and LOESS data (2099, red).

To investigate whether the QAPC method could be improved with normalization, we applied either LOESS normalization or sequence normalization in step 1 and compared the results with the results obtained from the raw data (Table 2). Data after (between microarray) LOESS normalization yielded similar results, but data after sequence normalization even better results (82 false positive, 67 false negative calls and error rate ∼0.22%). Quite similar high AUC values were obtained for all analyses. Hence, the normalization procedure does not seem to considerably affect the performance of the procedure. To further compare the normalization procedures, the absence-called probe sets were combined in a Venn diagram (Figure 4A). As expected, this resulted in a large intersection of 48 878 absence-called probes, and 1, 142 and 302 unique absence-called probes for the raw data, LOESS-normalized and sequence-normalized data respectively, as well as a relatively large overlap of 2099 between the raw data and LOESS normalized data. When analyzing the associated bit-scores of these probe sets, it became clear that the probe set with absence-called probes uniquely determined using sequence normalization had relatively low bit-scores compared to the other two probe sets (Figure 4B). This indicates that sequence normalization does improve the analysis. Altogether, these results indicate that using this method one can reliably make quantitative absence/presence calls in aCGH.

### Comparing genomes

**Figure 5.**
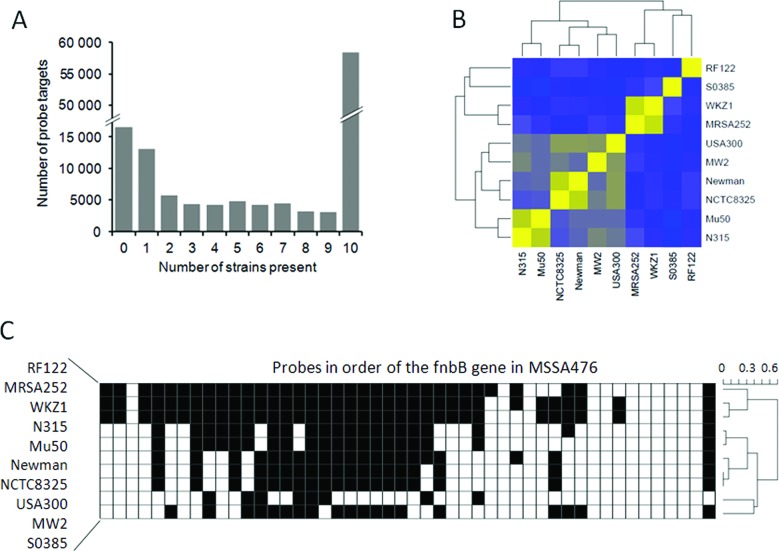
Comparing 10 *S. aureus* strains by QAPC. (**A**) Histogram of the number of probe targets per number of strains in which a probe target was called present. (**B**) Binary similarity matrix, based on the probes that were called present in of one these 10 strains. Yellow indicates a high similarity of 1 and blue a low similarity. (**C**) Plot indicating whether probe targets were called present (white) or absent (black) in the gene encoding fibronectin binding protein B (fnbB) based on the annotation of MSSA476.

Our QAPC method can be useful for application in comparative genomic analysis, such as to define core- and pan-genomes. As example, we compared 10 *S. aureus* strains: MRSA252, WKZ1, RF122, N315, Mu50, Newman, NCTC8325, USA300, MW2 and S0385. In this example, the genomic fragments of MSSA467 were used as positive-control target sequences. When the *P*-value cut-off is set to *P*(absent|intensity) ≤ 0.01, it was found that 16 457 probes do not have any target in any genome (Figure 5A). The overlap between all strains, the ‘core genome’, contained 58 381 target sequences. This example illustrates that the QAPC method enables the definition of a core genome without assigning a reference genome, to which all other strains are to be compared. The clustering clustered similarity matrix of the unique probe-target sequences (Figure 5B) very much resembles the phylogenetic results presented by (30). Our analysis confirmed that WKZ1–MRSA252, USA300–MW2, Newman–NCTC8325, Mu50–N315 are closely related pairs and strains RF122 and S0385 are relatively divergent.

This QAPC method generates absence/presence calls for individual probes rather than genomic regions. To show that this QAPC method can therefore be used for high-resolution aCGH analysis, we focused on the results for one gene: fibronectin binding protein B (fnbB). This gene is believed to be involved in pathogenesis. The fnbB gene is strikingly different in the different strains, only 11 probe targets are detected in all genomes (Figure 5C).

### Assessing NGS reads for *de-novo* assembly

**Figure 6.**
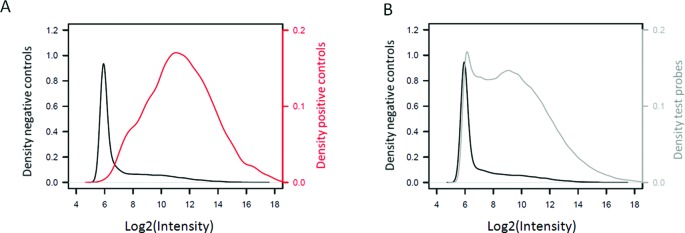
Evaluating NGS reads with the QAPC method. (**A**) Density plots showing the log2 signal intensities of the probes designed for the *A. gambiae* reference genome (red) and negative-control probes (black). (**B**) Density plots showing the log2 signal intensities of the negative-control probes (black) and the probes designed for the *C. riparius* NGS reads (grey).

Another application is the possibility to validate NGS reads in *de-novo* sequencing experiments of non-model organisms. As well documented, NGS genome sequencing suffers often from the presence of reads that do not belong to the sequenced sample. These cuckoo reads, which originate either from technical errors or sample contamination (31–33), hamper *de-novo* genome or transcriptome assembly. One solution is to validate the NGS sequence reads by aCGH using DNA of the concerned organism. As example, we validated the results of a *C. riparius de-novo* transcriptome sequencing experiment. For this, we designed a microarray for aCGH analysis with target probes for: the NGS read sequences (843 837), related species genbank sequences (75 984), as well as many negative controls (40 000). For this study, we included a positive-control genome in the experimental design; *A. gambiae* to determine the cut-off for the absence/presence calling. The results are shown in Supplementary Figure S2. It can be seen that many probes designed against *C. riparius* transcriptome reads indeed have generally a higher intensity in the *C. riparius* genome than in the *A. gambiae* genome. However, there are also many probes for which this is not the case. Prior to the data analysis we selected probes with a GC content below 60%. In total, 929 808 probes were included in the analysis, 23 036 of which were random negative controls and 9038 positive controls, as they matched the *A. gambiae* genome with a bit-score > 100. As the goodness of fit of the parametric distributions was poor, the analysis was based on density estimation with bandwidth optimization (cf. Supplementary Information). Again, the *A. gambiae* aCGH showed that the negative-control probes have a consistently lower intensity than the perfect-match probes, but with overlap (Figure 6A). Nevertheless, the AUC is 0.9081 which indicates that the intensity distributions are separated. We decided to discard probe targets only if they were called absent with a high certainty, thus we applied a (*P*(absent|intensity) > 0.9 cut-off to the *C. riparius* aCGH data, resulted in 169 273 (18.7%) absence-called probes that target NGS reads (Figure 6B). To explore NGS sample contamination as one explanation for the many absent reads, all NGS reads (1 458 164) from the *C. riparius* experiment were blasted against the human genome and transcriptome and 54 430 reads (3.7%) were found matching (bit-score > 95). In total, 102 121 (7.0%) probes were associated with these reads and could therefore be associated with human sample contamination. In total, 44 502 (44%) of these human-associated reads were called absent by our QAPC method and they constituted 26% of all absence-called probes. On the other hand, 55 619 of the 737 499 present probes were from reads matching human sequences. In other words, 26% of the absence-called probes were associated with human sequences, whereas this was only the case 7.8% of the remaining probes. The significant overrepresentation (Chi square test: X-squared = 49 204, *P*-value < 2.2e-16) of probes associated with human reads among the absence-called probes indicates that the QAPC method can be helpful in removing contaminating reads from NGS data.

## DISCUSSION

In this study, we propose a novel approach: the QAPC method for aCGH analysis that explicitly indicates the absence or presence of genomic sequences. The analysis procedure does not require any structural information of a reference genome, i.e. it does not require the physical order of the probe targets. This approach is therefore specifically useful for aCGH analyses on non-model organisms with poorly assembled genomes and/or limited genomic annotation. It should be noted that the order of the probe targets is conventionally included in the analysis to deal with error rates in copy number estimation. However, the QAPC method does not infer copy number, but specifically indicates whether probe targets are present or absent in the genome. Our raw results (Table 1) indicate that for such signals, which are relatively extreme, single probes can be informative. Encouraged by these results, we subsequently proposed a formal data analysis procedure that enabled a quantitative analysis for present or absent calling (Table 2).

Given that microarrays have been around for over 15 years now (34), we are not the first to present a method for absence/presence calling for probe targets. Traditionally, the majority of probe targets has been inferred by very precise probe (or feature) level modeling (e.g. (35)). The basis of these modeling approaches is that a certain signal-intensity level is estimated that should represent background noise and non-specific binding, and this value is subtracted from the total signal-intensity level of the feature. This subtraction approach has resulted in detection call algorithms in detail discussed by Schuster (36). The most well known is probably the MAS5 algorithm , based on a pairing of mismatch and perfect-match probes. Interestingly, most of the background noise and non-specific binding modeling efforts were made in the field of gene-expression analysis, rather than genomic (aCGH) analysis. It has proven to be hard to exactly quantify signal-intensity levels for probe-specific background noise and non-specific binding. Yet, the essence of the problem is that in gene-expression analysis it is difficult to claim that a gene is not expressed, if it cannot be measured. However, in aCGH analysis the relationship between signal intensity and genome content is less complex (2) and we have shown here that probes without genomic target, i.e. negative-control probes, in general have a consistently lower signal intensity than probes with target sequences (Table 1 and Figure 1). It is this consistent relationship that we use in our approach.

Given the above-mentioned difficulties, we decided stay clear from a signal-intensity subtraction method at the feature level. The basis of the QAPC method presented here is that total probe signal intensity is used as a proxy for the absence/presence of a probe target. We design a population of probes without and with target sequences in the biological sample, with various characteristics in terms of GC content, sequence, melting temperature and Gibbs free energy, all in a similar range as the target probes that are used for measuring the genomic content. We assume that the intensity of this set of negative-control and positive-control probes is a representative sample for the total signal-intensity range that probes without target can have in an experiment. In fact, this way of reasoning is not completely new. Zhang *et al.* (37) were interested in tissue-specific gene expression and called genes expressed if the probe intensity exceeded the 99th percentile of the intensities from 66 negative controls. In addition, the Illumina gene-expression platform contains negative controls to calculate a detection *P*-value for each gene. The number of negative-control probes ranges from 750 to 1600 for different types of BeadChips (different species and different versions). Shi *et al.* (38) proposed to use these probes to estimate the proportion of probe targets present in a sample, which is suggested as quality criterion for gene-expression analysis. We propose to use negative-control and positive-control probes in genomic (aCGH) analysis (instead of gene expression analysis) for probe target-specific absence/presence calling, taking into account the notion that probes with target sequences can have a low signal intensity (Table 1; Figure 1).

A novel feature of the QAPC method is the inclusion of positive-control probes. In principle, the method can be executed without explicitly testing positive controls, as could be dictated by specific experimental settings. This can be done by deconvolving a mixture of signals from probes without and with target sequences using an expectation maximization algorithm (albeit with strong distributional assumptions, see Supplementary Methods). However, it is advised to include a positive-control hybridization in the experimental design whenever possible. Not so much as reference for the genomic structure, which is usually the case in aCGH experiments, but rather for determining the signal intensity distribution of the positive-control probes. The positive-control hybridization can in principle be done with any genome for which sufficient sequence information is available. The analysis could benefit from choosing a positive-control genome that is to some extent biologically similar to the test genome, as this could make the hybridizations comparable.

Our QAPC method entails a direct comparison of the intensity values from the control hybridizations with the intensity values from the test genome. Thus, one assumption in the analysis is that the intensity values in both hybridizations are equivalent. This should be checked, for instance by comparing the intensities of the negative-control probes, or by including and comparing hybridization control spike-ins in both the test and the control sample. Another important assumption is that the distributions *P*(intensity|absent) and *P*(intensity|present) are well described. This can be checked with standard *q*–*q* plots. We supply a data analysis protocol that includes a decision scheme for normalization and model fitting (Supplementary Methods).

In *de-novo* non-model NGS experiments that by definition lack a reference genome, our method can be helpful to determine NGS-contaminating sequencing reads that do not belong to the genome of the sequenced organism. If probes generate a low QAPC call, this indicates that the probe is likely targeting a read, which is erroneous or present due to contamination of the DNA or RNA sample. Especially in RNA, probes can generate absence calls because they target a splice site or an RNA editing site. If necessary, additional bioinformatics analyses should be performed to analyze the probes with absent calls. For instance, a splice site in a read may be identified by a single absent called probe, contrasting the other probes associated with the read.

Apart from analyzing NGS reads, we have also shown that our QAPC method can be used to analyze regular aCGH experiments for instance to identify pan and core genomes (39). Structural information of a genome, i.e. the physical order of the probe targets, is not required for the data analysis, but it facilitates data plotting and interpretation, as was clear from our results for the fnbB gene of all *S. aureus* strains. It shows that the results give information about the absence/presence of single probe targets and therefore works at a resolution of a single probe. This can be useful for new aCGH experiments, but also for a reanalysis and reinterpretation of old experiments. Since aCGH experiments are performed with reference genomes, the QAPC method can in principle be used for re-analysis and re-interpretation of any previous experiment, if the arrays are fitted with negative-control probes. The data analyst only needs to ignore the genomic location of the probe targets and use the reference genome as positive control. In conclusion, given the presented applications and performance, like typical ROC AUC values larger than 0.9995, we advocate that the QAPC method can complement traditional aCGH analysis methods to enhance data interpretation.

## SUPPLEMENTARY DATA

Supplementary Data are available at NAR Online.

SUPPLEMENTARY DATA
