# Supplementary material for: Absence/presence calling in microarray-based CGH experiments with non-model organisms
Source: Nucleic Acids Res. 2014 Apr 25;42(11):e94. doi: 10.1093/nar/gku343 (PMC4066771; doi:10.1093/nar/gku343)
Supplement: SUPPLEMENTARY DATA [file supp_42_11_e94__index.html]

Absence/presence calling in microarray-based CGH experiments with non-model organisms — Absence/presence calling in microarray-based CGH experiments with non-model organisms — SUPPLEMENTARY DATA 

# Absence/presence calling in microarray-based CGH experiments with non-model organisms

## SUPPLEMENTARY DATA

**Files in this Data Supplement:**

- SUPPLEMENTARY DATA
