# Supplementary material for: Absence/presence calling in microarray-based CGH experiments with non-model organisms
Source: Nucleic Acids Res. 2014 Apr 25;42(11):e94. doi: 10.1093/nar/gku343 (PMC4066771; doi:10.1093/nar/gku343)
Supplement: SUPPLEMENTARY DATA [file supp_gku343_nar-00792-met-n-2014-File008.doc]

**- Supplementary Data –**

**Absence/presence calling in microarray-based CGH experiments with non-model organisms.**

Martijs J. Jonker ^1,2^, Wim de Leeuw^1,2^, Marino Marinković^1,3^, Floyd R.A. Wittink^1^, Han Rauwerda^1,2^, Oskar Bruning^1,2^, Wim A. Ensink^1^, Ad C. Fluit^4^ and C.H. Edwin Boel^4^, Mark de Jong^1^, and Timo M. Breit^1,2,*^

^1^ MicroArray Department & Integrative Bioinformatics Unit (MAD-IBU), Swammerdam Institute for Life Sciences (SILS), Faculty of Science (FNWI), University of Amsterdam (UvA), 1098 XH, Amsterdam, the Netherlands

^2^ Netherlands Bioinformatics Centre (NBIC), 6525 GA, Nijmegen, the Netherlands

^3^ Department of Aquatic Ecology and Ecotoxicology, Institute for Biodiversity and Ecosystem Dynamics (IBED), University of Amsterdam, Amsterdam, the Netherlands.

^4^ Medical Microbiology, University Medical Center Utrecht, Utrecht, the Netherlands

* To whom correspondence should be addressed.
Tel: +31-20-5257058; Fax: +31-20-5257762; Email: t.m.breit@uva.nl

Present Address: Dr. Timo M. Breit, MicroArray Department & Integrative Bioinformatics Unit (MAD-IBU), Swammerdam Institute for Life Sciences (SILS), Faculty of Science (FNWI), University of Amsterdam (UvA), Science Park 904, 1098 XH, Amsterdam, the Netherlands.

**ABSTRACT**

Structural variations in genomes are commonly studied by (micro)array-based comparative genomic hybridization (aCGH). The data analysis methods to infer copy number variation in model organisms (human, mouse) are established. In principle, the procedures are based on signal ratios between test and reference samples and the order of the probe targets in the genome. These procedures are less applicable to experiments with non-model organisms, which frequently comprise non-sequenced genomes with an unknown order of probe targets. We therefore present an additional analysis approach, which does not depend on the structural information of a reference genome, and quantifies the presence or absence of a probe target in an unknown genome. The principle is that intensity values of target probes are compared with the intensities of negative control probes and positive control probes from a control hybridization, to determine if a probe target is absent or present. In a test, analyzing the genome content of a known bacterial strain: *Staphylococcus aureus* MRSA252, this approach proved to be successful, demonstrated by receiver operating characteristic area under the curve values larger than 0.9995. We show its usability in various applications, such as comparing genome content and validating next-generation sequencing reads from eukaryotic non-model organisms.

Supplementary Table 1. Confusion table values to show the classification performance of negative control probes and perfect match probes when probe intensity is used as classifier in *Danio rerio*, when two times six replicate measurements and averaged probe intensities are used. The abbreviations indicate: TP: True positives, FP: False positives, TN: True Negatives, FN: False Negatives, TPR: True positive rate, FPR: False positive rate, TNR: True negative rate, FNR: False negative rate, P: Precision, AC: Accuracy, and AUC: Area under the curve

|  | *D. rerio* sample 1 | | | | | | *D. rerio* sample 2 | | | | | |  |
| --- | --- | --- | --- | --- | --- | --- | --- | --- | --- | --- | --- | --- | --- |
|  | repl. 1 | repl. 2 | repl. 3 | repl. 4 | repl. 5 | repl. 6 | repl. 1 | repl. 2 | repl. 3 | repl. 4 | repl. 5 | repl. 6 | mean signals |
| TP | 5,408 | 5,668 | 5,442 | 5,698 | 5,561 | 5,750 | 5,529 | 5,376 | 5,875 | 5,634 | 5,592 | 5,807 | 5,784 |
| FP | 1,503 | 1,224 | 1,337 | 1,454 | 1,376 | 1,164 | 1,348 | 1,423 | 1,309 | 1,501 | 1,140 | 1,241 | 1,293 |
| TN | 54,506 | 54,785 | 54,672 | 54,555 | 54,633 | 54,845 | 54,661 | 54,586 | 54,700 | 54,508 | 54,869 | 54,768 | 54,716 |
| FN | 1,363 | 1,103 | 1,329 | 1,073 | 1,210 | 1,021 | 1,242 | 1,395 | 896 | 1,137 | 1,179 | 964 | 987 |
| TPR | 0.7987 | 0.8371 | 0.8037 | 0.8415 | 0.8213 | 0.8492 | 0.8166 | 0.7940 | 0.8677 | 0.8321 | 0.8259 | 0.8576 | 0.8542 |
| FPR | 0.0268 | 0.0219 | 0.0239 | 0.0260 | 0.0246 | 0.0208 | 0.0241 | 0.0254 | 0.0234 | 0.0268 | 0.0204 | 0.0222 | 0.0231 |
| TNR | 0.9732 | 0.9781 | 0.9761 | 0.9740 | 0.9754 | 0.9792 | 0.9759 | 0.9746 | 0.9766 | 0.9732 | 0.9796 | 0.9778 | 0.9769 |
| FNR | 0.2013 | 0.1629 | 0.1963 | 0.1585 | 0.1787 | 0.1508 | 0.1834 | 0.2060 | 0.1323 | 0.1679 | 0.1741 | 0.1424 | 0.1458 |
| P | 0.7825 | 0.8224 | 0.8028 | 0.7967 | 0.8016 | 0.8316 | 0.8040 | 0.7907 | 0.8178 | 0.7896 | 0.8307 | 0.8239 | 0.8173 |
| AC | 0.9543 | 0.9629 | 0.9575 | 0.9597 | 0.9588 | 0.9652 | 0.9587 | 0.9551 | 0.9649 | 0.9580 | 0.9631 | 0.9649 | 0.9637 |
| AUC | 0.9760 | 0.9817 | 0.9782 | 0.9793 | 0.9779 | 0.9817 | 0.9793 | 0.9764 | 0.9827 | 0.9784 | 0.9812 | 0.9829 | 0.9823 |


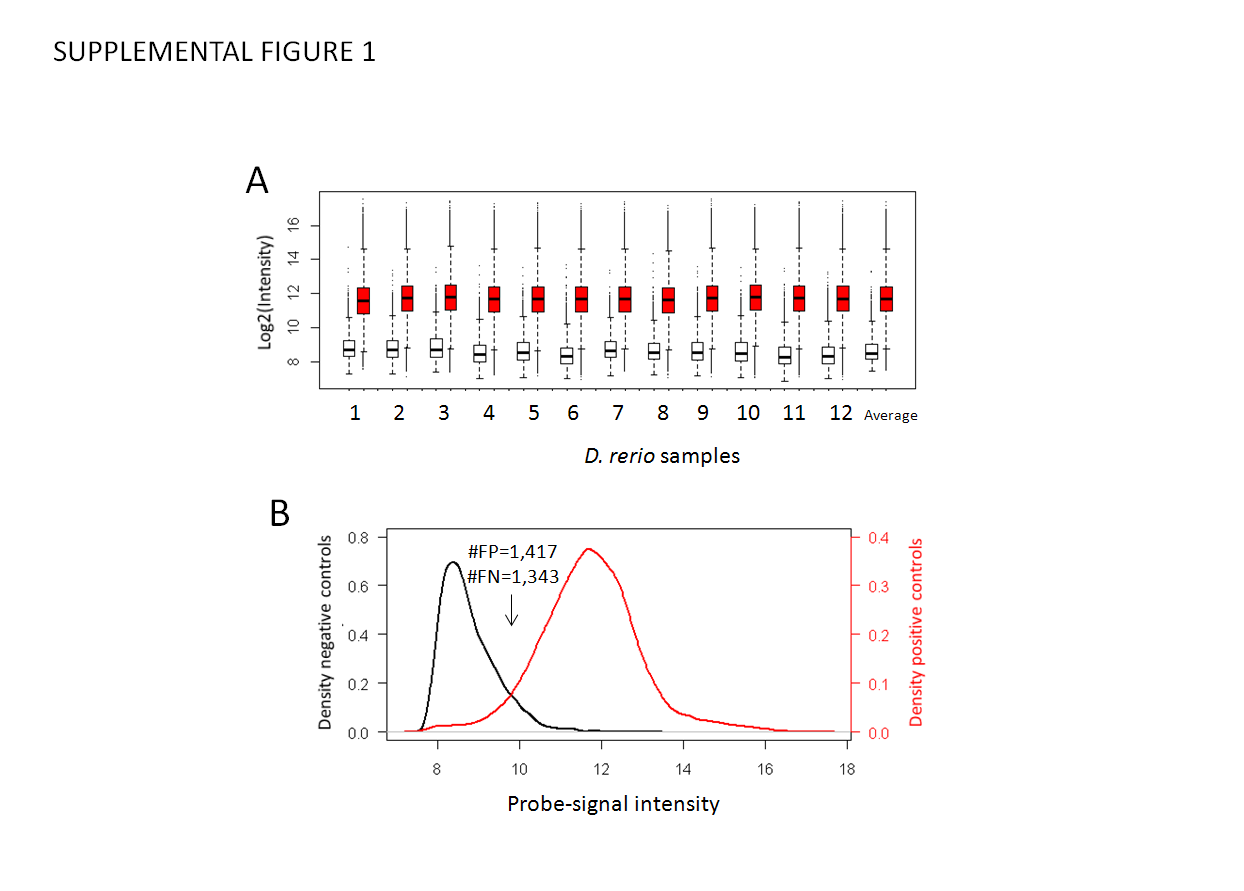


Supplementary Figure 1. A: box-and-whisker plots showing the intensity distributions of negative-control probes (white) and perfect-match probes (red) in twelve replicate *D. rerio* samples and based on the averaged signal. B: Density plot indicating the intensity distribution of the negative-control probes (black) and the perfect-match probes (red) of the averaged signal.


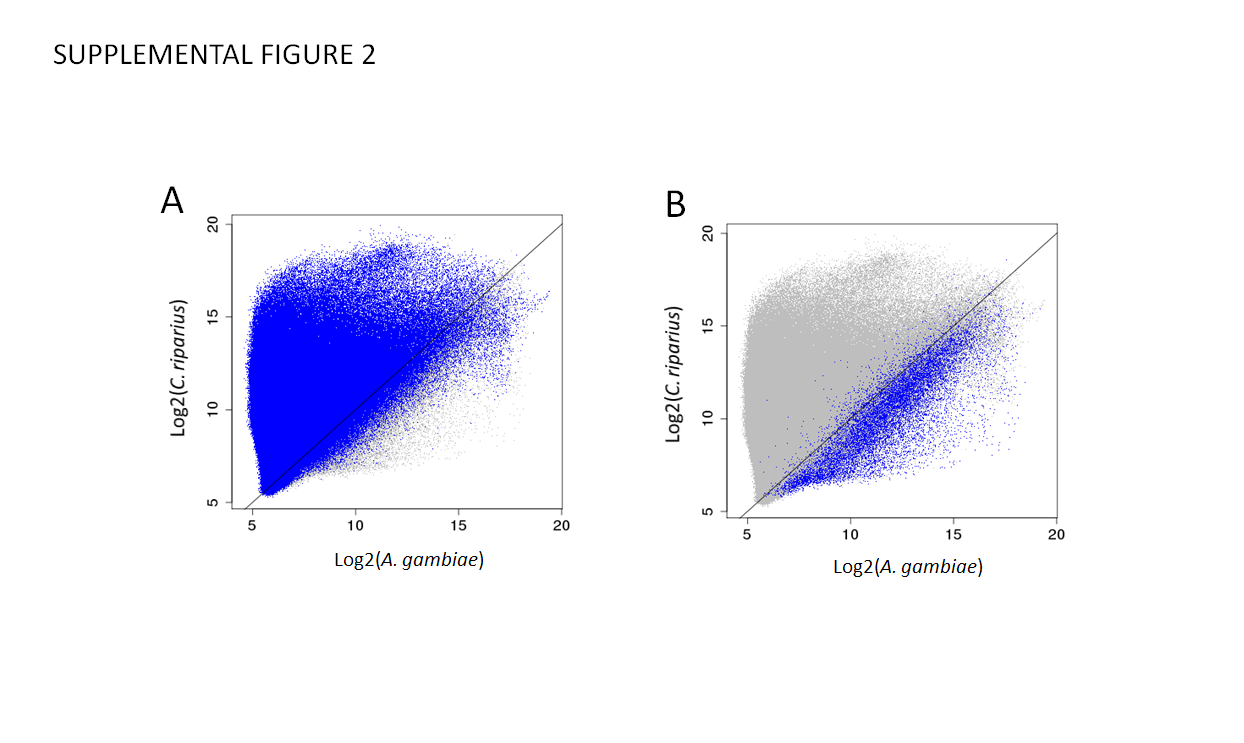


Supplementary Figure 2. The results for the *C. riparius* transcriptome validation microarray. The log2 probe intensity values after hybridizing *C. riparius* DNA is plotted against the log2 probe intensity values after hybridizing *A. gambiae* DNA. A: the values of the probes designed against the sequencing reads are indicated in blue. B: the values of the probes designed against the *A. gambiae* genome are indicated in blue.

**SUPPLEMENTARY METHODS**

The microarray based probe target detection method (QAPC method) is generally applicable and should work well on any dataset. Nevertheless, it is obviously required to perform a data quality control to assess whether the modeling procedures fit the data. There are two assumptions in the analysis that need specific attention.

**Assumption 1. The probe intensity values of the control and the test samples are equivalent.**

The QAPC method is based a direct comparison of the probe intensity values from the control hybridizations with the test hybridizations. Thus, one assumption in the analysis is that the intensity values in the two hybridizations are equivalent. In this study this was checked by plotting the intensities of the negative control probes and the intensities of the hybridization control spike-ins of the test sample(s) against the control samples. If a one-to-one relationship is observed, it is reasonable to assume that the intensities are equivalent. We also checked the density plots of the negative control probes. The densities should be similar in both the control and the test sample.

We did observe that the comparability of the probe intensities changed due the normalization procedures. The scatterplots should therefore be checked on pre- and post-normalized data. If the normalization procedure has a negative effect on the comparability of the probe intensity values of the control and the test sample, then the normalization procedure should be omitted, and inference is necessarily based on pre-normalized data.

In the paper three normalization steps are discussed, which may need some further explanation. Each normalization step should improve the quality of the data by removing technical biases. The first normalization step entails between and/or within slide normalization, which is an established procedure for microarrays. The second step is the correction of probe sequence effects, which can improve the analysis as intensities of probes with different sequences are compared. The third normalization step is an adjusted quantile normalization, which should make the intensities of the test and control sample comparable. In principle, all three normalization steps do make sense intuitively, and they worked well on the test data set. We have nevertheless observed that each normalization procedure can influence the comparability of test and control sample, and not all normalization procedures work well in every experiment. For instance, we did observe that the sequence normalization and the quantile normalization had a negative effect on the comparability of the *C. riparius* and *A. gambiae* hybridization signals. Possibly because *C. riparius* was measured with Cy3 dye and *A. gambiae* with Cy5 dye, on the same array. This was done for reasons of costs, but a more reliable analysis was probably performed when the hybridizations were performed on different arrays with the same Cy-dye, or with dye-swaps. Thus, the usual considerations for microarray experimental design apply here also. All in all, for normalizing the data, we do suggest to use the decision flow diagram shown in Supplementary Figure 3.

**Assumption 2. The modeled distributions of p(intensity|absent) and p(intensity|present) fit the observed intensity distributions of the negative- and positive control probes.**

The calculation of the QAPC call depends on the quantification of p(intensity|absent) and p(intensity|present). Thus, an important assumption is that the distributions p(intensity|absent) and p(intensity|present) are well quantified. We have tested various options to model the intensity distributions of the positive and negative control probes. We observed that the negative control probes can frequently be described by the shifted log normal distribution or the normal (Gaussian) distribution. The positive-control probes can frequently be described by a normal distribution. This can easily be checked with q-q plots, based on the percentiles of the data. We used the percentiles of the data, because outliers had a large influence on the inference of the q-q plot if the individual data points were used. If these empirical distributions showed a lack of fit we used a density estimation procedure with bandwidth optimization for conditional probabilities. This procedure was computationally intensive and not practical for large data sets. The procedure was usable up to 3,000 data points, i.e., 3,000 positive- and negative-control probes. Our experiments usually contained many more positive and negative control probes, and we applied a sampling procedure to reduce the data set and calculate approximate conditional p-values. We did not apply a random sampling procedure, as we wanted to keep the densities of the probe intensities intact. Rather, we sampled each 5^th^ percentile of the distribution for *n_sample_*/*n_total_* of the data points in this bin, where *n_sample_* indicates the number of positive and negative controls we need to sample (e.g. for positive controls typically 3000**n_pos_*/(*n_pos_*+*n_neg_*) ), and *n_total_* indicates the total number of present or absent calls. As in this procedure control data points are omitted, which means information loss, we decided to use the parametric distributions when possible.

Normalize using standard QC

Are test and control comparable?

Keep the normalized data

Yes

No

Skip the normalization

Sequence based normalization

Are test and control comparable?

Keep the normalized data

Quantile based normalization

Yes

No

Skip the normalization

Are test and control comparable?

Keep the normalized data

Calculate QAPC calls

Yes

No

Skip the normalization

Supplementary Figure 3. Flow diagram for choosing the suitable normalization procedure(s) for the QAPC method

**Data analysis strategies used on the datasets in the paper.**

Following the guidelines mentioned above, the datasets in the paper were analyzed with the following methods:

A) *S. aureus* data (Figure 5):

1. Sequence based normalization
2. Adjusted quantile normalization
3. Negative control probe intensities are modeled using a Gaussian distribution.
4. Positive-control probe intensities are modeled using a Gaussian distribution.

A) *C. riparius and A. gambiae* data (Figure 6):

1. Standard loess normalization using the negative control probes and hybridization control probes only, to alleviate the dye bias
2. Sequence and quantile normalization were omitted
3. Negative- and positive-control probe intensities are modeled using density estimation with bandwidth optimization.

**Data analysis without using a positive control hybridization.**

It may sometimes be not possible to incorporate a positive control hybridization in the experiment. In general, we observed that the probe intensities of the test hybridization showed bimodal distributions. (Figure 2 and Figure 6 in the paper). This suggests that the distribution of intensities consist of a mixture of signals from probes interrogating sequences that were present in the sample, and probes interrogating sequences that were absent in the sample. A naïve approach would be to simply model the distributions of signals as a mixture of two Gaussians, which is solved using expectation maximization (EM). However, if it is known that the negative control probes have no targets in the test sample (and if positive-control probes do have targets), then it would improve the analysis if this information could be incorporated in the analysis. The following procedure is suggested.

The logarithm of the likelihood that EM maximizes is:

$$\lambda\left(X;\theta\right)=\sum_{n=1}^{N} \log\sum_{k=1}^{K} p_{k}g\left(\mathbf{x}_{n};m_{k};\sigma_{k}\right)$$

for *N* probes (features) and *K* classes (usually two: absent or present probe targets). The mixing probabilities are indicated as *p_k_* and *g* indicates a gaussian distribution with mean *m* and standard deviation *σ*. Prior knowledge about the control probes could be incorporated using a indicator vector *I*, which indicates whether *x_n_* is based on a negative control (*I_n_*=1), a positive control (*I_n_*=2), or whether the class is unknown (*I_n_*=3). The mixing probabilities *p_k_* can then be defined as as *p_k_*(*I_n_*). If *x_n_* represents the intensity of a negative control probe, then *p*_1_(*I_n_*) = 1 and *p*_2_(*I_n_*) = 0. The values are reversed if *x_n_* represents the intensity of a positive-control probe and *p_k_*(*I_n_*) needs to be estimated if *x_n_* is a regular data point. The logarithm of the likelihood

$$\lambda\left(X;\theta\right)=\sum_{n=1}^{N} \log\sum_{k=1}^{K} p_{k}\left(I_{n}\right)g\left(\mathbf{x}_{n};m_{k};\sigma_{k}\right)$$

can now be maximized with the E step:

$$p^{\left(i\right)}\left(k|x_{n},I_{n}\right)=\frac{p_{k}^{\left(i\right)}\left(I_{n}\right)g\left(x_{n};m_{k}^{\left(i\right)};\sigma_{k}^{\left(i\right)}\right)}{\sum_{k=1}^{K} p_{k}^{\left(i\right)}\left(I_{n}\right)g\left(x_{n};m_{k}^{\left(i\right)};\sigma_{k}^{\left(i\right)}\right)}$$

and the M step

$$m_{k}^{\left(i-1\right)}=\frac{\sum_{n=1}^{N} p^{\left(i\right)}\left(k|x_{n},I_{n}\right)\mathbf{x}_{n}}{\sum_{n=1}^{N} p^{\left(i\right)}\left(k|x_{n},I_{n}\right)}$$

$$\sigma_{k}^{\left(i-1\right)}=\sqrt{\frac{\sum_{n=1}^{N} p^{\left(i\right)}\left(k|x_{n},I_{n}\right)\left(x_{n}-m_{k}^{\left(i-1\right)}\right)^{2}}{\sum_{n=1}^{N} p^{\left(i\right)}\left(k|x_{n},I_{n}\right)}}$$

$$p_{k}^{\left(i-1\right)}\left(I_{n}=3\right)=\frac{1}{N^{*}}\sum_{n=1}^{N} p^{\left(i\right)}\left(k|x_{n},I_{n}=3\right)$$

which is not much different from the standard procedure for mixed Gaussians. Yet, the mixing probabilities for *I_n_*=1 and *I_n_*=2 are predefined, and can be excluded from the M step, and hence *N** is the number of unknown data points. In this formulation, the mixing probabilities *p_k_*(*I_n_*) and therefore the membership probabilities *p*(*k*|*x_n_*, *I_n_*) are now directly indicating membership to the negative or positive controls, and the associated values *x_n_* contribute to the parameter estimation of the distribution of the correct class. In addition, the conditional p-values are now equivalent to the conditional p-values generated by analyses with a positive control hybridization. Obviously, it is assumed that the intensity distributions of probes with and without targets in the sample can be described by normal distributions.
